# Risky Preferences: Nursing Staff Perceptions of Risks May Impede Delivery of Person-Centered Care

**DOI:** 10.1093/geroni/igaa057.3065

**Published:** 2020-12-16

**Authors:** Liza Behrens, Kimberly Van Haitsma, Ann Kolanowski, Marie Boltz, Mark Sciegaj, Katherine Abbott, Caroline Madrigal

**Affiliations:** 1 University of Pennsylvania School of Nursing, Philadelphia, Pennsylvania, United States; 2 Penn State University, University Park, Pennsylvania, United States; 3 PSU, University Park, Pennsylvania, United States; 4 Miami University, Oxford, Ohio, United States; 5 Providence VA Medical Center, Providence, Rhode Island, United States

## Abstract

Nursing home (NH) staff perceptions of risks to residents’ health and safety is a major barrier to honoring resident preferences, the cornerstone of person-centered care delivery. This study examined direct-care nursing staff perceptions of risk (possibilities for harm or loss) associated with honoring residents’ preferences for everyday living and care activities. Participants (N=27) were mostly female (85%), had more than 3 years of experience (74%), and worked in NHs experiencing 6-12 health citations. Content analysis of 12 focus groups indicated nursing staff perceptions of risks may impede delivery of PCC. This is supported by the overarching theme: pervasive risk avoidance; and sub-themes of: staff values, supports for risk-taking, and challenges to honoring preferences. Findings will be discussed considering a newly modified risk engagement framework meant to understand and inform the clinical management of older adult preferences perceived to carry risks. Opportunities for future research will be discussed (e.g. measurement development). Part of a symposium sponsored by the Research in Quality of Care Interest Group.

